# Characterizing Treatment-Resistant Anorexia Nervosa

**DOI:** 10.3389/fpsyt.2020.542206

**Published:** 2021-01-08

**Authors:** Sarah Smith, D. Blake Woodside

**Affiliations:** ^1^Department of Psychiatry, University of Toronto, Toronto, ON, Canada; ^2^Department of Psychiatry, University Health Network, Toronto, ON, Canada

**Keywords:** anorexia nervosa, eating disorder, inpatient, treatment resistant, premature termination of treatment

## Abstract

**Background:** The issue of treatment resistance in eating disorder care is controversial. Prior research has identified multiple failed treatment attempts as a common criterion for severe and enduring anorexia nervosa, but little is known about patients who have multiple failed treatment attempts. This study was designed to compare the clinical and demographic characteristics of eating disorder patients with multiple, incomplete inpatient admissions to those with good outcomes. Understanding if these patient populations differ at initial admissions has implications for the prediction and characterization of inpatient eating disorder treatment resistance.

**Methods:** This study analyzed existing data from a specialist inpatient eating disorder program at a large Canadian teaching hospital collected between 2000 and 2016. Treatment resistance was defined as two or more incomplete admissions and no complete admissions in the study period. Data were available on 37 patients who met this criteria, and 38 patients who had completed their first admission and remained well (defined as a BMI > 18.5 with no binging or purging behavior) 1 year after discharge. Variables of interest included age, weight, diagnoses, duration of illness, eating disorder psychopathology, eating disorder behavioral frequencies and depressive symptoms at the time of index inpatient admissions. Statistical analyses consisted of Mann–Whitney U tests, Chi-square tests, and a logistic regression.

**Results:** In our main bivariate analyses, patients with multiple incomplete admissions were characterized by more severe eating disorder psychopathology and depressive symptoms at admission as well as an increased prevalence of the binge purge subtype of anorexia nervosa. In our exploratory multivariate analyses controlling for diagnostic subtype and depressive symptoms, severity of eating disorder psychopathology did not remain significant. No statistically significant difference in body mass index (BMI) or frequencies of eating disorder behaviors were found. A trend toward a longer duration of illness did not meet statistical significance.

**Conclusions:** This study found that patients considered resistant to inpatient eating disorder treatment differ from those with good outcomes at initial admission. These results suggest that while treatment-resistant anorexia nervosa may be related to severe and enduring anorexia nervosa, it may also be a different concept that warrants additional research.

## Introduction

Anorexia nervosa is considered one of the most difficult psychiatric disorders to treat (1). This eating disorder is characterized by severe restriction of food intake resulting in significantly low body weight, an intense fear of gaining weight and undue influence of body weight or shape in self-evaluation (2). Anorexia nervosa often has its onset in childhood or adolescence (3) and despite treatment, 20–25% of patients develop a chronic form of the illness (4, 5).

There has been an increasing focus on potential treatment options for longstanding eating disorders in recent years with a growing body of research on the concepts of chronic eating disorders (6) and severe and enduring anorexia nervosa (SE-AN) (7). These terms are often used interchangeably (6), with a prolonged length of illness (i.e., >7–10 years) as their most common defining criteria (7). Severity itself is defined by the Diagnostic and Statistical Manual of Mental Disorder 5th Edition (DSM-5) in terms of body mass index (BMI), although it allows the inclusion of clinical symptoms, functional disability, and supervision requirements in this assessment (2). There is also a growing body of literature on the concept of treatment resistance, which is often considered another component of severe and enduring anorexia nervosa. A recent review by Broomfield et al. (7) identified a history of multiple failed treatment attempts as the second most common criteria in published definitions of SE-AN, although what constituted a failed treatment attempt, and the number of failed attempts required to meet this criterion, was not clear across studies. Indeed, there is no established definition of treatment-resistant anorexia nervosa (8, 9).

Prior studies on inpatient care have conceptualized treatment resistance as patterns of multiple admissions to hospital (9, 10) or readmissions to specialist eating disorder services (11). For patients who are severely medically compromised or who have not benefitted from outpatient care, inpatient eating disorder care is the most intensive form of treatment available (12). For these patients, inpatient treatment provides a structured environment, supervision, and medical monitoring (13). Many specialist inpatient programs also provide multidisciplinary care and psychotherapy (12). Despite this, inpatient eating disorder programs have high rates of premature termination of treatment, or dropout, ranging from 20% to 51% (14) and rates of readmission ranging from 27% to 42% (15, 16). Prior research at the site of this study reported premature treatment termination rates of 36–51% over time (17–19) with a higher prevalence of the binge-purge subtype diagnosis among patients who did not complete treatment (defined as achieving a BMI of 20 kg/m^2^) (17, 19). Studies of potential predictors of premature termination of inpatient treatment at other sites have reported mixed results on the effect of patient diagnoses (18–22), age at admission (19–22), duration of illness (19–22), body mass index at admission (19–22), eating disorder beliefs and cognitions (18–22), eating disorder behavior frequencies (i.e., binging and purging) (19–22), and depressive symptoms (19–22). However, across studies, patients who do not complete inpatient eating disorder treatment consistently have shorter lengths of stay (18, 19, 21) and are discharged at lower body weights than patients who complete treatment (18, 21, 23).

These findings are of high clinical relevance as patients who leave treatment at low body weights are more likely to remain symptomatic after discharge, suffer severe depressive symptoms and be readmitted to specialist inpatient eating disorder care (15). However, almost nothing is known about patients who have multiple incomplete admissions: those who can be considered resistant to specialist inpatient eating disorder care. The purpose of this study was to explore the characteristics of these patients at one specialist inpatient eating disorder unit compared to patients admitted in the same time period who completed treatment and remained well 1 year after discharge.

### Research Question and Hypotheses

This study was designed to compare the characteristics of eating disorder patients with anorexia nervosa with multiple incomplete inpatient admissions (two or more) and no complete admissions to patients with good inpatient treatment outcomes in a retrospective study. Drawing from prior research on treatment resistance in inpatient eating disorder care that defined treatment resistance as multiple incomplete (or failed) treatment attempts, we considered patients with two or more incomplete inpatient admissions and no complete admissions as resistant to specialist, inpatient care.

Based on this research, research on premature termination of inpatient eating care and our clinical experience, we defined the following hypotheses: (i) Patients who have multiple incomplete admissions to our specialized inpatient eating disorder unit will have longer lengths of illness than those who have good outcomes, and (ii) Patients who have multiple incomplete admissions to our specialized inpatient eating disorder unit will be more likely than those who have good outcomes to have the binge-purge subtype of anorexia nervosa. As additional variables of interest have yielded mixed results in studies of premature termination of inpatient treatment or readmission, their inclusion was considered exploratory, and no *a priori* hypotheses were established.

## Methods and Materials

### Participants

**Figure 1:**
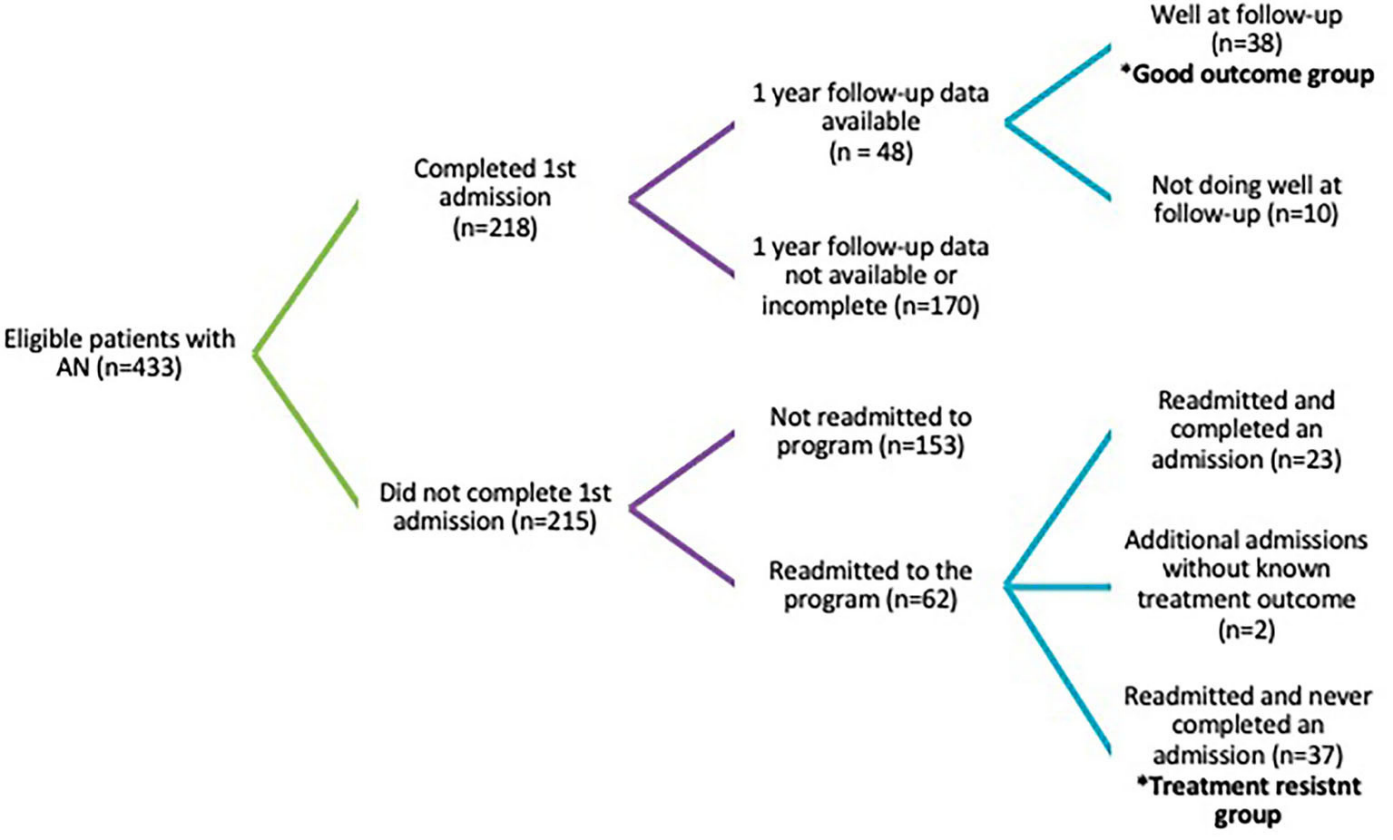
Treatment outcomes of patients admitted to TGH August 2000 to August 2016.

This study is a secondary analysis of data on 75 patients admitted to the inpatient eating disorder unit at the Toronto General Hospital between January 2001 and December 2015. All patients met the DSM-IV diagnostic criteria for anorexia nervosa according to the Diagnostic and Statistical Manual of Mental Disorders, Fourth Edition, Text Revision (DSM-IV-TR) at the time of admission and consented to participate in research. Data was collected between August 2000 and August 2016. Data was available on 433 patients with anorexia nervosa who consented to participate in research, of whom 37 (8.5%) had multiple incomplete admissions and no complete admissions, while 38 (8.8%) completed their first admission and remained well and available for follow-up for 1 year (Figure 1). Patients were considered to have a good outcome if they remained well at 1-year follow-up, defined as maintaining a BMI >18.5 in the absence of binging or purging in the 3 months preceding. Patients were considered to be treatment resistant if they had had two more incomplete admissions and no complete admissions in the study period. Patients with multiple incomplete admissions as well as admissions without known outcomes were not included in the treatment-resistant group. These two patient subgroups represent the best and worst outcomes observed during the study period. As all patients offered admission to the inpatient program at Toronto General Hospital have serious eating disorders and are often medically unstable, these subgroups were chosen to magnify potential differences in a superficially quite homogenous patient population. All analyses were of admission data from patients' first admission during the study period.

### Intervention

During the period these data were collected, the inpatient eating unit program at the Toronto General Hospital was an intensive program that focused on medical stabilization, weight restoration, the normalization of eating behaviors, psychosocial rehabilitation, and group therapy. As it was one of the few specialized adult eating disorder units in its province, it admitted patients from a large geographical area. All patients were admitted voluntarily. Treatment was provided by an interdisciplinary team that included psychiatrists, psychologists, dieticians, social workers, nurses, and occupational therapists. As patients progressed in the program, they were granted more privileges with the goal of transitioning to day attendance. Patients were considered to have completed the inpatient program when they were medically stable and had attained a minimum body mass index (BMI) of 20 kg/m^2^. Upon completion of the inpatient program, some patients transitioned to an affiliated day hospital program, while others choose to pursue other forms of outpatient care independently. Inpatient treatment could also be terminated before treatment completion by the patient or treatment team. Patients could choose to discharge themselves from the unit at any time if they were medically stable or they could be discharged by staff for not participating in the program. Staff initiated premature terminations of treatment typically involved long periods of discussion with the patient about their difficulties participating in the program and unsuccessful attempts to modify their behaviors before discharge.

### Measures

All patients completed a standardized battery of psychological measures at the time of admission including components of the Eating Disorder Examination (EDE), an Eating Disorder Examination Questionnaire (EDEQ), a Beck Depression Inventory (BDI), and demographic questions. For patients who had multiple admissions in the study period data from their first admission was used for analyses.

The EDE is a semi-structured diagnostic interview that assesses concerns about weight, shape, diet, and the of frequency eating disorder behaviors in the 3 months preceding administration (24). The EDE is a common tool in eating disorder research and has been shown to have good internal consistency (Cronbach α = 0.67–0.79) (25) and interrater reliability (*r* = 0.69–1.00) (26). In this study, data from the EDE was used to examine the frequency of the eating disorder behaviors of binge eating, self-induced vomiting (purging), and exercise before admission.

The Eating Disorder Examination Questionnaire (EDEQ) is a 28-item self-report questionnaire that is used to assess eating disorder beliefs and cognitions (27). It has four calculated subscales (shape concern, weight concern, eating concern, and restraint) and a total score. In this study, only the total score was used for analyses given our small sample size. The EDEQ has been shown to have good internal consistency (Cronbach's α = 0.78–0.93) and test–test reliability (*r* = 0.81–0.94) (28).

The Beck Depression Inventory (BDI-II) is a 21-item self-report questionnaire of depressive symptoms that has a calculated total score (29). It one of the most commonly used research measures of depression and has been used in prior studies to measure depressive symptoms in eating disorder patients (19–22). The BDI has been shown to have high internal consistency (Cronbach α > 0.75) (23).

Height and weight were measured at admission and were used to calculate the body mass index (BMI). Weight was then measured weekly until discharge. This information was used to calculate weight gain in inpatient treatment, rate of weight gain, and BMI at discharge. Duration of illness was self-reported by patients at the time of admission.

### Statistical Analyses

Statistical analyses were conducted in SPSS Version 24 (SPSS Inc, Chicago). Chi-square tests were used for categorical variables of interest (gender, diagnostic subtype, living situation, and employment status). Continuous variables of interest (age at admission, duration of illness, BMI at admission, BDI total score at admission, EDEQ total score at admission, frequency of eating disorder behaviors in the 3 months preceding admission, length of inpatient treatment in weeks, weight gain in treatment, rate of weight gain in treatment, and BMI at discharge) were examined for normality. As almost all were not normally distributed, non-parametric Mann–Whitney U tests were used for between-group analyses. A Bonferroni correction was applied to correct for multiple comparisons resulting in an alpha level of 0.003.

Significant variables in the univariate analyses were selected for inclusion in an exploratory binary logistic regression to control for potential confounding. Given the relatively small sample size, the number of variables eligible for inclusion was reduced based on clinical significance to avoid overspecification. Multicolinearity of the final model was assessed using the variance inflation factor with a reference value of four. More complex modeling was not possible due to the small sample size.

## Results

All patients (*N* = 75) met the criteria for DSM-IV anorexia nervosa at the time of admission with 46 meeting the criteria for the restricting subtype (AN-R) (61.3%) and 29 meeting the diagnostic criteria for the binge-purge subtype (AN-BP) (38.7%). Patients ranged in age from 17 to 62 years old (*M* = 25.92; SD = 8.35) at admission and had been unwell for an average of 6.70 years (SD = 6.78). Patients' BMIs at admission ranged from 11 to 18 kg/m^2^ (*M* = 14.83, SD = 1.73). All patients reported their gender identity, with 97.3% identifying as female and 2.7% identifying as male. Sixty-six patients (88%) reported their employment status, of whom 26 (39.4%) were employed preceding admission, 25 were attending school (37.9%), and 15 were unemployed (22.7%). Sixty-five (86.7%) patients reported their living circumstance of whom 9 lived independently (13.8%), 39 lived with their family of origin (60.0%), 15 lived with partners and/or their children (23.1%), and two lived with roommates (3.1%).

During the study period, 37 patients had two or more incomplete inpatient admissions and no complete admissions. These patients were classified as resistant to inpatient treatment. In this period, 38 patients completed their first admission and remained well (defined as maintaining a BMI >18.5 kg/m^2^ without of binging and purging for at least 3 months) at 1-year follow-up. These patients were classified as having good outcomes.

Treatment-resistant patients were more likely to have the binge purge subtype of anorexia nervosa than those who had good outcomes [χ^2^ (1, *n* = 75) = 16.99, *p* < 0.001]. Treatment-resistant patients did not differ statistically from patients who had good outcomes in terms of gender, occupation, age, or BMI at admission (Table 1). There was a trend of differences in employment status between treatment-resistant patients and those with good outcomes [χ^2^(2, *n* = 66) = 6.15, *p* = 0.046). *Post hoc* testing of residuals showed that this was due to a greater percentage of patients being unemployed in the treatment-resistant group (34.5%) than the good outcome group (10.8%, *p* = 0.02).

**Table 1:** Demographics of treatment-resistant patients and patients with good outcomes at admission.

**Variable**	***N***	**Treatment resistant % or median (IQR)**	**Good outcome % or median (IQR)**	**Test of significance**
Diagnosis	75			*x*^2^(1) = 16.99, *p* < 0.001
AN-R		37.8%	84.2%	
AN-BP		62.2%	15.8%	
Gender	75			*x*^2^(1) = 2.00, *p* = 0.157
Female		100%	94.7%	
Male		0%	5.3%	
Occupation	66			*x*^2^(2) = 6.15, *p* = 0.046
Employed		37.9%	40.5%	
Student		27.6%	48.6%	
Unemployed		34.5%	10.8%	
Living Situation	65			*x*^2^(3) = 0.688, *p* = 0.876
Independent		13.8%	13.9%	
Parents		55.2%	63.9%	
Partner/children		27.6%	19.4%	
Roommates		3.4%	2.8%	
Age	74	24.00 (21.50, 30.00)	22.00 (20.00, 27.00)	*U* = 556.50, *p* = 0.167
Duration of illness in years	69	6.84 (2.62, 12.32)	2.87 (1.46, 9.43)	*U* = 440.50, *p* = 0.068
BMI in kg/m^2^	75	14.36 (13.56, 15.60)	15.59 (13.85, 16.38)	*U* = 558.00, *p* = 0.124
EDEQ total score	55	5.29 (4.76, 5.59)	4.57 (3.35, 5.17)	*U* = 179.00, *p* = 0.003
BDI total score	57	46.00 (34.00, 53.00)	33.00 (21.00, 40.50)	*U* = 159.00, *p* < 0.001
Weekly # days of excessive exercise in 3 months before tx	69	2.80 (0, 6.63)	4.00 (0, 7.00)	*U* = 544.50, *p* = 0.553
Weekly # of binge episodes in 3 months before tx (BP subtype)	23	0.17 (0, 6.67)	0.00 (0, 0.33)	*U* = 40.0, *p* = 0.406
Weekly # of purge episodes in 3 months before tx (BP subtype)	24	7.33 (2.83, 28.08)	5.17 (0, 11.33)	*U* = 38.50, *p* = 0.300

Treatment-resistant patients scored significantly higher on the BDI (Mdn = 46.00) than patients who had good outcomes (Mdn = 33.00, *U* = 159.00, *p* < 0.001). Similarly, treatment-resistant patients had significantly higher EDEQ total scores (Mdn = 5.29) than patients who had good outcomes (Mdn = 4.57, *U* = 179.00, *p* = 0.003), although they did not differ significantly in their report of eating disorder behavior frequencies including binge eating, purging, or excessive exercise in the 3 months preceding admission (Table 1). There was a trend for treatment-resistant patients to have longer durations of illness (Mdn = 6.84) than those with good outcomes (Mdn = 2.87, *U* = 440.50, *p* = 0.068), but this did not achieve statistical significance.

Treatment-resistant patients had shorter first admissions to the inpatient unit in weeks (Mdn = 4.29) than patients with good outcomes (Mdn = 16.43, *U* = 51.00, *p* < 0.001). They also gained less weight as inpatients (Mdn = 4.50 kg) compared to those with good outcomes (15.25, *U* = 48.00, *p* < 0.001). Thus, treatment-resistant patients were discharged from the program with lower BMIs (Mdn = 16.23) than those with good outcomes (Mdn-20.57, *U* = 1.00, *p* < 0.001), although their weekly rate of weight gain while admitted to the inpatient unit did not differ statistically (Mdn = 0.73) from those with good outcomes (Mdn = 0.90, *U* = 585.00, *p* = 0.284) (Table 2).

**Table 2:** Treatment outcomes of treatment-resistant patients and patients with good outcomes.

**Variable**	***N***	**Treatment resistant median (IQR)**	**Good outcome median (IQR)**	**Test of significance**
Median weeks of treatment received	75	4.29 (3.14, 7.71)	16.43 (14.14, 18.57)	*U* = 51.00, *p* < 0.001
Median BMI at discharge in kg/m^2^	74	16.23 (14.80, 17.62)	20.57 (20.36, 21.04)	*U* = 1.00, *p* < 0.001
Median weight gain to discharge in kg	74	4.50 (2.10, 7.65)	15.25 (10.80, 18.60)	*U* = 48.00, *p* < 0.001
Median weekly rate of weight gain from admission to discharge in kg	74	0.73 (0.51, 1.22)	0.90 (0.77, 1.05)	*U* = 585.00, *p* = 0.284

Independent variables that were significant in bivariate analyses (EDEQ total score, BDI total score, weeks of treatment, weight gain in treatment, and BMI at discharge) were then considered for inclusion in an exploratory logistic regression based on clinical significance. Potential correlations between these variables were assessed using Spearman correlations (Table 3). As admission BMIs and rate of weight gain did not differ significantly between groups in bivariate analyses, discharge BMI and total weight gain were both considered to be a function of length admission in weeks. To avoid potential overspecification, only BMI at discharge was included in the model in addition to diagnostic subtype, EDEQ total score, and BDI total score.

**Table 3:** Spearman correlations between significant variables in univariate analyses.

	**Diagnostic** **subtype**	**Discharge** **BMI**	**Weight** **gain**	**Weeks in** **treatment**	**BDI score** **(admission)**	**EDEQ score** **(admission)**
Diagnostic subtype	1.00	−0.45^**^	−0.41^**^	−0.40^**^	0.35^**^	0.29^**^
Discharge BMI		1.00	0.82^**^	0.75^**^	−0.29^*^	−0.17
Weight gain			1.00	0.89^**^	−0.40^**^	−0.41^**^
Weeks in treatment				1.00	−0.35^**^	−0.28^*^
BDI score at admission					1.00	0.66^**^
EDEQ score at admission						1.00

* p < 0.05;

** *p < 0.01*.

Analyses of the initial model showed complete separation of data points limiting interpretability. Independent variables were then examined individually, which showed that discharge BMI fully discriminated between groups. To allow the assessment of other independent variables, this variable was removed from the model resulting in a final model with three independent variables associated with group membership (subtype, BDI total score, and EDEQ total score at admission). This model was found to be statistically significant, χ^2^(3) = 21.70, *p* < 0.001, with no evidence of multicolinearity. Examination of independent variables within the model showed that the binge purge subtype of anorexia nervosa and higher BDI scores remained statistically significant. Both of these independent variables were associated with higher odds of having an eating disorder resistant to inpatient treatment (Table 4). However, having a higher EDEQ total score did not retain statistical significance when controlling for subtype and BDI score.

**Table 4:** Patient characteristics associated with multiple, incomplete admissions to inpatient care without any complete admissions in a logistic regression model.

**Independent variables**	**Adjusted OR (95% CI)**	**Test statistic**	***p*-Value**
Omnibus Likelihood Ratio		21.70 (3)	*p* < 0.001
Subtype (AN-BP vs. AN-R)	4.60 (1.05, 20.15)	4.09	*p* = 0.04
BDI total score	1.09 (1.01, 1.18)	4.61	*p* = 0.03
EDEQ total score	1.29 (0.47, 3.51)	0.25	*p* = 0.62

## Discussion

The results of this study show that treatment-resistant patients comprised approximately 10% of the patients treated in our specialist, adult inpatient eating disorder program between 2000 and 2016. This finding is concerning as a specialist, inpatient eating disorder treatment is the most intensive form of adult eating disorder treatment available in Canada and patients who are unable to benefit from it have few other options for treatment.

These treatment-resistant eating disorder patients who were not able to complete multiple specialist, inpatient admissions differed from those who were able to complete their first admissions and remain well after treatment. Specifically, eating disorder patients considered resistant to inpatient treatment were more likely to have a diagnosis of anorexia nervosa binge purge subtype, present with more severe depressive symptoms, and endorse more severe eating disorder beliefs and cognitions (psychopathology) despite not presenting at lower body weights or reporting engaging in higher frequencies of specific eating disorder behaviors in bivariate analyses. Patient diagnostic subtype and severity of depressive symptoms remained significant predictors of treatment resistance in an exploratory multivariate model controlling for severity of eating disorder psychopathology as measured by the EDEQ. These findings suggest that it is possible to identify patients at high risk of repeated, incomplete admissions at the time of their initial admission. These findings also represent a novel contribution to eating disorder research as no prior studies have characterized patients with repeated, incomplete admissions to specialist, inpatient care.

Many of the characteristics of patients considered treatment resistant in this study are the same features associated with repeat admissions to inpatient eating disorder care. Specifically, our findings that patients who do not complete inpatient treatment have shorter lengths of stays and are discharged at lower body weights are consistent with prior research on readmission (18, 19, 21). However, our finding in bivariate analyses that treatment-resistant patients differ in their eating disorder cognitions and depressive symptoms compared to those with positive treatment outcomes contrasts the findings of prior research that has compared patients requiring multiple admissions (often defined as two admissions) to inpatient eating disorder care to those singular admissions with mixed results (20–22). This could be explained by our group selection criteria as our definition of treatment resistance, two more incomplete admissions, and no complete admissions, likely magnified group differences and made it possible to detect differences obscured when patients with multiple admissions but differing treatment outcomes were examined as a homogenous group.

Similarly, as our definition of treatment resistance included multiple incomplete inpatient admissions, several of the characteristics that differentiated our treatment-resistant group from those with good outcomes have been previously associated with premature termination of treatment. Specifically, the binge purge subtype of anorexia nervosa (18–20), more severe eating disorder cognitions (18–20), and more severe depressive symptoms (19) have been associated with one episode of premature treatment termination, although these findings have not been consistent across studies of premature termination of treatment (20–22). Again, it is possible that our definition of treatment resistance may have amplified group differences not detectable when patients who completed their admissions were compared to those who terminated only one admission prematurely in prior studies.

In contrast, we did not find that patients with multiple incomplete admissions differed statistically from those with good outcomes in body mass index or frequency of eating disorder behaviors at initial admission. Indeed, at admission, the median BMI in both of our outcome groups fell in the severe or extreme categories of severity in DSM-IV. This is likely a reflection of the patient population served by the inpatient eating disorder at Toronto General Hospital as patients admitted to this specialized program were typically medically very unwell or had not benefitted from outpatient care. Preceding admission, the majority of patients in both outcome groups resided with their families of origin making it unclear whether they were unable to live independently. Similarly, while not statistically significant, a larger proportion of patients in the treatment resistant outcome group were unemployed suggesting that they were not able to work independently, while a larger proportion of patients in the good outcome group were students prior to admission. These findings may speak to the functional impairment of severe eating disorders and their potential impact on patients' lives.

Statistically, patients in our two outcome groups also did not differ in terms of age at admission, but a trend was seen in the length of illness with patients having two or more incomplete admissions having longer durations of illness than those with positive outcomes. Visual inspection of the median durations of illness in these groups (Table 1) suggests that this trend may be clinically significant but did not achieve statistical significance due to our relatively small sample size. This may represent a Type 2 error. If this is the case, then our study would be consistent with prior research that has reported an association between longer durations of illness and poor treatment outcomes (21) as well as definitions of severe and enduring anorexia nervosa that include multiple incomplete or failed treatment approaches (7). If this is not the case, then our findings support the understanding that patients with severe and enduring anorexia nervosa may be a heterogeneous population wherein some patients have had recurrent, incomplete treatment attempts, while others have not. The potential relationship between duration of illness and treatment resistance remains unclear, as many patients may not have had access to specialist eating disorder services or chosen not to seek care preceding their referral to our program. In our study, duration of illness was defined as the length of illness preceding patients' first admission to intensive, inpatient treatment but whether their admission was necessitated by the severity of their symptoms, the failure of outpatient treatment, or both, is unknown. Future research should attempt to quantify what proportion of patients with severe and enduring anorexia nervosa have had recurrent incomplete trials of treatment, and what these specific treatments have consisted of, to inform whether severity of symptoms and length of illness are associated with treatment outcomes when controlling for prior incomplete treatment trials. As prior treatment attempts have not been included in all definitions of severe and enduring anorexia employed in prior studies, it is possible that there are patients in this population who have not had prior trials of intensive treatment for whom such care would still be appropriate to trial.

Interestingly, the results of our exploratory multivariate analyses found that severity of eating disorder cognitions did not predict resistance to inpatient treatment when controlling for the effect of depressive symptoms and diagnostic subtype. One possible interpretation of this finding is that severe depressive symptoms may impede engagement in treatment. It is also possible that patients with more severe depressive symptoms reported more severe eating disorder symptoms as many symptoms of depression such as impaired concentration, excessive guilt, and negative self-evaluation overlap with eating disorder symptomology making it difficult to distinguish their etiology.

Regardless, the findings of this study identify a subset of severely ill eating disorder patients for whom the most intensive form of adult eating disorder treatment, specialist inpatient care is currently not effective in achieving nutritional rehabilitation and weight restoration. Based on prior operational definitions of treatment resistance as multiple incomplete or failed treatment attempts, we have considered these patients resistant to the specialist, inpatient eating disorder program where this study was conducted. It is possible that these patients may have benefitted from a different program or approach. Given the lack of a consistent definition of treatment resistance across treatment settings, we also propose that treatment resistance should be further explored on a program or approach-specific basis in future research to inform the development of a more global and generalizable definition of treatment resistance in eating disorder treatment.

### Limitations

Limitations of this study include our relatively small sample size and the selection of our sample. The data used for this study was collected over a 16-year period at one of the few specialized, inpatient eating disorder programs in Canada and one of the largest. In this time, only 37 (8.5%) patients who consented to participate in research had multiple, incomplete admissions and no complete admissions, the worst treatment outcome in this study. It is possible that other patients had subsequent incomplete inpatient admissions to other programs that were not captured. Similarly, of 218 patients who completed their first admission, follow-up data was only available for 48 patients (Figure 1). It is possible that other patients were also doing well and either receiving care somewhere other than the Toronto General Hospital or not requiring specialist outpatient care. The implication of this small sample is that we may not have had adequate power to detect relevant significant differences between treatment-resistant patients and those with a good outcome. The limited number of patients in our regression analyses may also explain why severity of eating disorder psychopathology was not significant in multivariate analyses. Furthermore, while the choice to compare patients with multiple incomplete admissions to those with good outcomes was made to magnify potential differences between patient subgroups, our findings may not represent differences between patients with treatment-resistant eating disorders and all patients seeking inpatient eating disorder care. Finally, the patients in this study all required specialized, inpatient treatment, the most intensive form of adult eating treatment available in Canada. While this allowed for a study on resistance to inpatient eating disorder care, results may not be generalizable to patients not requiring such intensive care.

## Conclusion

Anorexia nervosa remains a difficult illness to treat. Almost 10% of patients treated in our specialist, inpatient eating disorder program for anorexia nervosa over a 15-year period had two or more incomplete admissions and no complete admissions. These treatment-resistant patients represent a severely ill subset of patients who were not able to achieve nutritional rehabilitation and weight restoration in a specialist inpatient treatment and who differed clinically from patients with good outcomes in the same program. Additional research is required to better characterize the clinical characteristics and health service use of these patients to inform the development of eating disorder treatments that could better meet their needs.

## Data Availability Statement

The datasets generated for this study are available on request to the corresponding author.

## Ethics Statement

The studies involving human participants were reviewed and approved by Research Ethics Board, University Health Network. The patients/participants provided their written informed consent to participate in this study.

## Author Contributions

SS and DW contributed to the conception and design of the study. SS completed the literature review, organized the database, completed the statistical analyses under DW's supervision, and wrote the first draft of the manuscript. All authors contributed to the manuscript revisions.

## Conflict of Interest

The authors declare that the research was conducted in the absence of any commercial or financial relationships that could be construed as a potential conflict of interest.
